# Is a shortened postoperative albendazole duration after curative surgery for alveolar echinococcosis possible? Results from a prospective multicenter study

**DOI:** 10.1186/s13071-025-07095-1

**Published:** 2025-11-17

**Authors:** Paul Calame, Carine Richou, Oleg Blagosklonov, Frederic Grenouillet, Alexandra Heurgué, Isabelle Villena, Emilia Frentiu, Celia Turco, Gabriel Simon, ECHINOVISTA Group, Alexandre Doussot, Sophie Felix, Florence Grenouillet, Bruno Heyd, Damien Montange, Claire Vanlemmens, Pascal Chavanet, Frédéric Dalle, Sandrine Gohier, Anne Minello, Jérôme Dumortier, Martine Wallon, Anne Debourgogne, Valérie Laurent, Lorraine Letranchant, Marie Machouart, Jérôme Watelet, Cathy Chemla, Thomas Feron, Christine Hoeffel, Daniele Sommacale, Gerard Thiéfin, Dominique-Angèle Vuitton, Solange Bresson-Hadni

**Affiliations:** 1 Department of Radiology, University Marie and Louis Pasteur and University Hospital Besançon, 25030 Besançon, France; 2 National Reference Center for Echinococcoses, Department of Parasitology-Mycology, University Hospital Besançon, 25030 Besançon, France; 3 Department of Hepatology, University Marie and Louis Pasteur and University Hospital Besançon, 25030 Besançon, France; 4 Department of Nuclear Medicine, University Marie and Louis Pasteur and University Hospital Besançon, 25030 Besançon, France; 5 Laboratory of Fungi and Parasite Serology, University Marie and Louis Pasteur and University Hospital Besançon, 25030 Besançon, France; 6 University Marie and Louis Pasteur, CNRS, Chrono-Environment Research Unit, 25030 Besançon, France; 7 Department of Gastroenterology and Hepatology, University Hospital of Reims, 51092 Reims, France; 8 Department of Parasitology, University Hospital of Reims, 51092 Reims, France; 9 Department of Infectious Disease, University Hospital of Nancy, 54511 Vandoeuvre-Lès-Nancy, France; 10 Department of Digestive Surgery, University Marie and Louis Pasteur and University Hospital Besançon, 25030 Besançon, France; 11 School of Health Sciences, University Marie and Louis Pasteur, 25030 Besançon, France

**Keywords:** Alveolar echinococcosis, *Echinococcus multilocularis*, Curative surgery, Albendazole, rEm18-ELISA

## Abstract

In a prospective study of 15 patients with liver alveolar echinococcosis who underwent curative surgery, a strategy to reduce the recommended 2-year postoperative albendazole (ABZ) regimen was evaluated. Guided by negative positron emission tomography computed tomography (PET-CT) and magnetic resonance imaging (MRI) results at 1 year, ABZ was safely discontinued earlier (median 14 months) in 10 patients, with no recurrence observed during the mean 47 months of follow-up, indicating that postoperative ABZ may be safely shortened with careful imaging follow-up. Interestingly, a retrospective analysis of the rEm18AB antibody index showed that a negative index was not required for ABZ withdrawal, although a declining trend supported the decision.

**Graphical Abstract:**

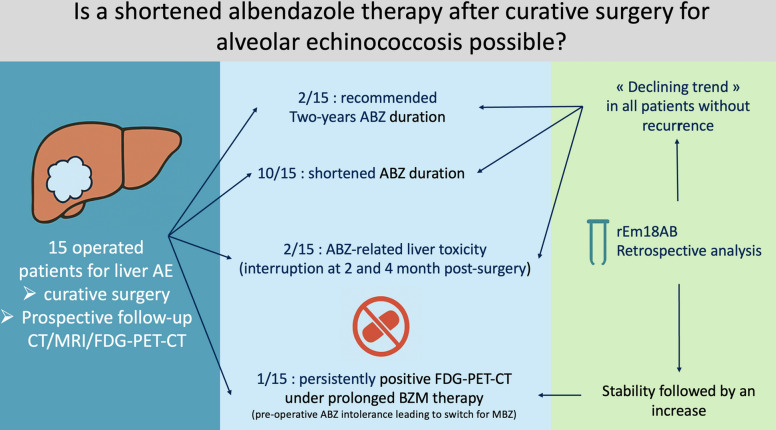

## Background

Alveolar echinococcosis (AE) is a rare life-threatening parasitic disease caused by the larval stage of *Echinococcus multilocularis* [1]. It predominantly affects the liver as an infiltrative, tumor-like lesion. The mainstay of treatment is the complete surgical resection of the lesion (“curative surgery”), in combination with albendazole (ABZ) therapy for 2 years, a duration which was established by the World Health Organization (WHO) international consensus [2], provided that no recurrence was identified by conventional liver imaging [1–3]. However, ABZ treatment is burdened by potential hepatotoxicity and leukopenia [1, 4], which necessitates dose reduction or discontinuation. Furthermore, its teratogenicity requires effective contraception in women of childbearing age. Therefore, evidence-based approaches that could safely shorten ABZ therapy duration are needed. The use of 18F-fluorodeoxyglucose (18F-FDG) positron emission tomography combined with computed tomography (PET-CT) to assess the perilesional metabolic activity of the AE lesion may help identify patients in whom ABZ could be discontinued without risk of recurrence [1, 3]. Measurement of antibodies against recombinant Em18 (rEm18AB), which correlate with parasite viability, has emerged as a promising tool for assessing treatment efficacy [5]. However, prospective data supporting ABZ withdrawal before the standard 2-year mark are lacking. In addition, it remains unclear whether the rEm18AB index normalizes after 2 years of ABZ in surgically cured patients without imaging recurrence.

Outcomes in a prospective cohort of surgically treated AE patients who underwent personalized ABZ therapy guided by concurrent PET-CT and magnetic resonance imaging (MRI) findings were evaluated, associated with a retrospective rEm18AB index analysis.

## Methods

### Study population

This analysis used data from the prospective multicenter EchinoVISTA study (NCT02876146), conducted in six university hospitals in eastern France between June 2012 and July 2016 [6]. The primary objective of the EchinoVISTA study was to evaluate the utility of combining PET-CT with conventional imaging for guiding timely ABZ withdrawal in patients with hepatic AE. A secondary objective, addressed herein using prospectively collected serum samples, was to investigate how monitoring the rEm18AB index with a commercial assay could inform treatment decisions. The study protocol adhered to European and French regulations and received approval from relevant ethics committees (CPP-Est2 #11/606) and data protection authorities (CNIL [French National Commission on Informatics and Liberty] approval). All participants provided written informed consent after receiving detailed information regarding study objectives, procedures, risks, and benefits. Eligibility for the main EchinoVISTA study required available baseline imaging (including PET-CT), AE serology, and complete clinical data. For the present sub-analysis focusing on postoperative management, inclusion was restricted to patients who underwent surgical resection with curative intent.

CT, MRI, and FDG PET-CT were performed according to standard institutional liver protocols; PET-CT included early (1 h) and delayed (3 h) acquisitions [7]. Post-surgical imaging was scheduled every 6 months for the first 2 years and annually thereafter. Imaging and clinical data were prospectively collected during scheduled multidisciplinary team (MDT) follow-up boards.

PET-CT negativity required no subjective perilesional FDG uptake on both 1 h and 3 h acquisitions, confirmed by a lesion-to-liver maximum standardized uptake value (SUVmax) ratio ≤ 1.0, and MRI negativity required no new or progressive AE microcysts, allowing only stable postoperative changes.

All imaging data (CT, MRI, PET-CT) were systematically prospectively reviewed during dedicated AE MDT meetings and in a non-blinded manner. These meetings involved expert review of CT and MRI scans by radiologists with specific expertise in AE, and review of PET-CT scans by nuclear medicine physicians experienced in AE assessment. The MDT composition also included hepatologists, parasitologists, clinical biologists, and specialized hepatobiliary surgeons. Based on the comprehensive review of imaging findings alongside clinical and biological data within these MDT meetings, consensus decisions regarding the continuation, dosage adjustment, or withdrawal of ABZ therapy were made according to the study protocol.

Postoperative ABZ was administered continuously at 15 mg/kg/day in two divided doses (typically 400 mg twice daily; maximum 800 mg/day), with regular laboratory monitoring. Therapeutic drug monitoring of ABZ sulfoxide 4 h after morning intake (peak level) was used to guide dose adjustment (target: 1–3 µmol/L) [8].

### Serological analyses (rEm18AB detection)

Blood samples for serological analysis were systematically collected before surgery, 1 month post-surgery, every 3 months for the first 2 years post-surgery, and then annually, or more frequently if clinically indicated. While prospective serological monitoring included the commercial Em2+ enzyme-linked immunosorbent assay (ELISA; Bordier Affinity Products, Crissier, Switzerland), the specific analysis of rEm18 antibodies (rEm18AB) was performed retrospectively on serum aliquots stored at −80 °C in the EchinoVISTA biobank. Measurements utilized the commercial rEm18-ELISA^®^ kit (Bordier Affinity Products, performance [in-lab]: repeatability 4.9–7.5%; reproducibility 12.0–18.3%). Results were expressed as a numerical index (reported to two decimal places). As per manufacturer guidelines [9], an index value ≥ 1 was classified as positive, and < 1 as negative. Given the retrospective assessment of rEm18AB, ABZ withdrawal decisions were not influenced by serology.

## Results

Among the 45 patients included in the EchinoVISTA study, 16 (36%) underwent curative surgical resection of AE liver lesions (12 men, 4 women, 54 ± 17 years old at diagnosis). None of them was immunosuppressed. The median maximal AE lesion diameter was 6.5 cm (interquartile range [IQR]: 5.7; 7.9 [range: 3.8–11]), predominantly in the right liver lobe (75%). Thirteen (81%) patients were classified as P2N0M0 and three (19%) as P3N0M0 [10]. Median time from diagnosis to surgery was 121 days (IQR: 81; 263 [range: 60–720]), with a median preoperative ABZ duration of 3.5 months (IQR: 2; 6.5 [range: 1–18]). The liver resections consisted of right hepatectomy (*n* = 9), left hepatectomy (*n* = 3), mesohepatectomy (*n* = 1), posterior sectionectomy (*n* = 1), segment V segmentectomy (*n* = 1), and left lateral sectionectomy (*n* = 1). Negative margins (R0) were achieved in 9/16 (56%), whereas an R1 resection, defined by a microscopic margin ≤ 1 mm, was obtained in 7/16 (44%). Following one immediate postoperative death, 15 patients were monitored for a mean of 46.7 months (IQR: 36; 60 [range: 24–72]). ABZ therapy was discontinued due to liver cytolysis in two patients at 2 and 4 months postoperatively, respectively; neither patient had a recurrence during the 5-year follow-up.

**Fig. 1:**
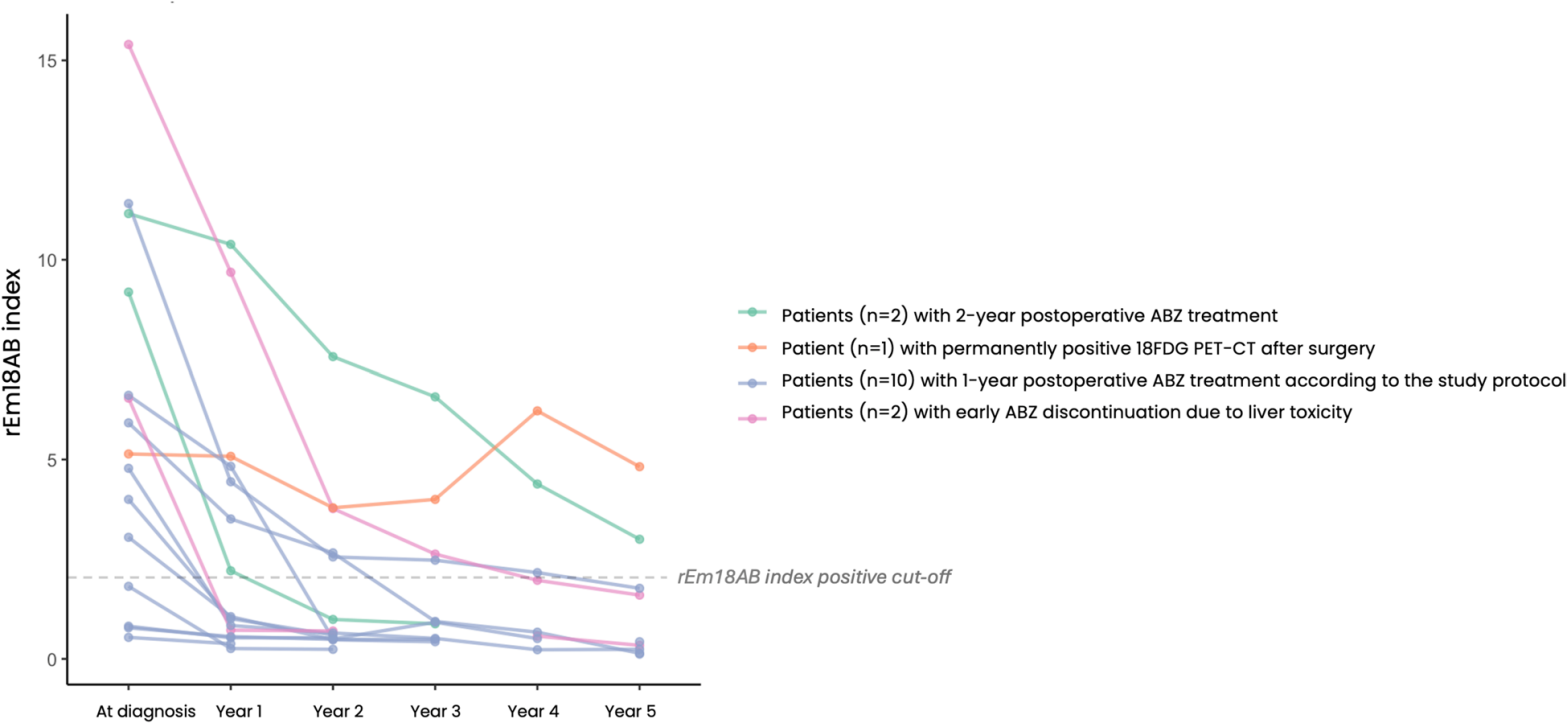
Postoperative course of the rEm18AB index after curative resection of the liver alveolar echinococcosis lesions in 15 patients. The patient who died in the immediate postoperative period was excluded. The patient with permanently positive 18F-FDG PET-CT received mebendazole because of albendazole liver toxicity

Based on the EchinoVISTA protocol, ABZ therapy was electively shortened in 10 patients (median duration: 14 months; IQR: 12.3; 17.3 [range: 12–18]). This decision was triggered by the absence of metabolic activity on PET-CT and negative morphological imaging (MRI) at 1 year post-surgery. None of these 10 patients experienced recurrence. At the time of ABZ withdrawal, five had a negative rEm18AB index, while five exhibited markedly decreasing, yet still positive (≥ 1), indices (Fig. 1).

Two patients continued ABZ for the standard 2 years despite negative imaging at 1 year, guided by persisting anti-Em2+ antibody levels. Both still had rEm18AB indices ≥ 1 when ABZ was stopped after 2 years of treatment, and neither relapsed.

One patient, in whom ABZ therapy was switched to mebendazole pre-surgery due to ABZ toxicity, required ongoing antiparasitic treatment. This was due to persistent FDG uptake on PET-CT at 1 h and 3 h near the resection site during the first 2 years post-surgery (despite negative MRI) and a non-declining rEm18AB index throughout the 5-year follow-up.

In the 14 patients without recurrence at the end of follow-up, the median decrease in rEm18AB from its baseline value was 50.8% (IQR: 31; 72 [range: 7–86]). At the last study follow-up, the rEm18AB index remained positive in 1/10 (10%) patients with protocol-driven early withdrawal, 1/2 (50%) with withdrawal at 2 years, and 1/2 (50%) with early withdrawal due to toxicity, all without recurrence. The estimated rEm18AB half-life was 18 months (IQR: 11; 27 months).

## Discussion

While ABZ remains an integral part of AE management, its patient-dependent bioavailability, which may vary with time, and its potential toxicity underscore the importance of minimizing unnecessary exposure. It is worth noting that severe ABZ-related liver toxicity was observed in 3/15 patients (i.e., 20%) in our cohort. This study is the first prospective investigation specifically designed to evaluate ABZ therapy shortening after curative AE surgery. Our findings, based on a careful 48-month follow-up, demonstrate that, in patients with no recurrence proved by MRI and PET-CT at 1 year after surgery, discontinuation of ABZ earlier than the currently recommended 2-year period can be achieved without risk. In this study, rEm18AB serum levels were not part of the decision criteria to shorten ABZ administration after surgery, but were studied retrospectively for their possible contribution to the treatment decision. In half of those patients with early ABZ withdrawal and no recurrence, the rEm18AB index was still ≥ 1 when the decision was made to discontinue ABZ. Notably, in one patient with ABZ discontinued 4 months after surgery because of liver toxicity, the rEm18AB index, very high at diagnosis, was still high at 1 year; similar observations were made for the two patients with ABZ withdrawn 2 years after surgery, according to the international consensus [3]. But in all these cases, there was no recurrence, which introduces the concept that a declining trend—rather than an absolute negative index—may serve as a complementary key-point for shortening treatment duration. Waiting for a negative index should not be the absolute goal, because it may unduly prolong treatment. Interestingly, both patients in whom the treatment was withdrawn at 2 years after surgery, according to the international consensus [3], still had an elevated rEm18 index at that time, which decreased over the subsequent years of follow-up. Thus, unlike the algorithm proposed by Gloor et al. [11], postoperative decision-making for stopping ABZ at 1 year should not require a serological threshold—or even seronegativity—as it would needlessly prolong ABZ postoperative exposure. Absence of morphological and metabolic recurrence associated with rEm18AB declining index after 1 year of treatment would justify ABZ withdrawal. Conversely, stable rEm18AB index levels and/or abnormalities on PET-CT or MRI would justify continuation or resumption of ABZ therapy. Patients who had a very elevated rEm18AB index before surgery had the longest persistence of antibodies against the *E. multilocularis* viability marker Em18. The half-life of immunoglobulins and possible prolonged polyclonal activation of B lymphocytes, long after the resection of all parasite tissue, may explain such an observation.

Recurrence after curative surgery in patients with AE is rare, with reports of 1/88 (1%) [11] and 2/74 (2%) [12] in the literature. In the retrospective study by Gloor et al. [11], the prognostic value of the continuous decrease or re-increase in rEm18ABs levels was also stressed. Notte et al. [12], however, observed that the safety margin status of the surgical resection (R0 or R1) was not associated with recurrence. Consistent with this observation, the only recurrence in our cohort occurred after an R0 resection. Accordingly, the margin status should not drive decisions to abbreviate or prolong postoperative ABZ. Decisions should be guided primarily by standardized imaging criteria, namely MRI and FDG PET-CT at 1 year, and careful long-term follow-up (i.e., 10 years according to the 2010 WHO guidelines [2]), which may include rEm18AB measurement as confirmation, should be ensured for all patients.

This study has limitations. First, it lacks a concurrent 24-month control group and relies on historical practice for comparison. In addition, despite its multicenter design, the number of included patients is small, and generalizability to Chinese cohorts is limited given differences in lesion size and time to surgery [13], thus warranting larger collaborative studies—including Western China centers—to evaluate safe postoperative ABZ shortening in those populations.

In conclusion, these encouraging findings support considering earlier discontinuation of ABZ after curative surgery when cross-sectional imaging and FDG PET-CT are negative and serological kinetics are favorable, while calling for larger, protocol-harmonized cohorts with longer follow-up to confirm these results and refine postoperative ABZ management in AE.

## Data Availability

Data supporting the main conclusions of this study are included in the manuscript.

## Abbreviations

AE: Alveolar echinococcosis
ABZ: Albendazole
rEm18AB: Recombinant Em18 antibodies
18F-FDG PET-CT: 18-Fluorodeoxyglucose positron emission tomography computed tomography
MDT: Multidisciplinary team
MRI: Magnetic resonance imaging
CT: Computed tomography
